# Critical appraisal of the role of volumetric modulated arc therapy in the radiation therapy management of breast cancer

**DOI:** 10.1186/s13014-017-0935-4

**Published:** 2017-12-19

**Authors:** Luca Cozzi, Frank Lohr, Antonella Fogliata, Davide Franceschini, Fiorenza De Rose, A R Filippi, Gabriele Guidi, Valentina Vanoni, Marta Scorsetti

**Affiliations:** 1 Radiotherapy and Radiosurgery Department, Humanitas Research Hospital, Via Manzoni 56, 20089 Rozzano-Milan, Italy; 2 Department of Biomedical Sciences, Humanitas University, Rozzano, Italy; 3 Radiation Oncology Department, Ospedaliero-Universitaria, Modena, Italy; 4 Radiation Oncology Department, Osp. S. Chiara, Trento, Italy; 5 Medical Physics Department, Az. Ospedaliero-Universitaria, Modena, Italy; 6 Department of Radiation Oncology, Osp. S. Luigi Gonzaga University Hospital, Torino, Italy

**Keywords:** Volumetric modulated arc therapy, Breast cancer, Radiotherapy

## Abstract

**Background:**

The aim of this review is the critical appraisal of the current use of volumetric modulated arc therapy for the radiation therapy management of breast cancer. Both clinical and treatment planning studies were investigated.

**Material and methods:**

A Pubmed/MEDLINE search of the National Library of Medicine was performed to identify VMAT and breast related articles. After a first order rejection of the irrelevant findings, the remaining articles were grouped according to two main categories: clinical vs. planning studies and to some sub-categories (pointing to significant technical features). Main areas of application, dosimetric and clinical findings as well as areas of innovations were defined.

**Results:**

A total of 131 articles were identified and of these, 67 passed a first order selection. Six studies reported clinical results while 61 treatment dealed with treatment planning investigations. Among the innovation lines, the use of high intensity photon beams (flattening filter free), altered fractionation schemes (simultaneous integrated boost, accelerated partial breast irradiation, single fraction), prone positioning and modification of standard VMAT (use of dynamic trajectories or hybrid VMAT methods) resulted among the main relevant fields of interest. Approximately 10% of the publications reported upon respiratory gating in conjunction with VMAT.

**Conclusions:**

The role of VMAT in the radiation treatment of breast cancer seems to be consolidated in the in-silico arena while still limited evidence and only one phase II trial appeared in literature from the clinical viewpoint. More clinical reports are needed to fully proove the expected dosimetric benefits demonstrated in the planning investigations.

## Background

The introduction of volumetric modulated arc therapy (VMAT) in clinical practice dates back to 2008 after the publication of the seminal work of Otto [1] which opened the road to the implementation of VMAT optimisation algorithms in the treatment planning systems. Since then, VMAT has been applied to almost all possible clinical indications and a huge amount of research was published. The navigation through this plethora of articles is challenging and for this reason, the availability of site-specific reviews might help to appraise the state of the art and the role of VMAT in the management of the radiation oncology process. Breast cancer is one of the most common diseases and its incidence is increasing and will continue to increase due to early diagnosis programs as well as to the demographic effect.

Radiotherapy is a fundamental component of the multidisciplinary approach to breast cancer and, depending on several factors, many different fractionation schemes and treatment modalities have been applied and explored with success. The clinical use of VMAT for breast cancer is still limited, according to published data, but it is potentially a versatile solution, applicable to whole breast or partial breast treatments, conventional or altered fractionation schemes (sequential or with simultantous integrated boost, hypofractionated and even in single fraction). Also from the technological point of view, interesting variants of VMAT have been proposed and tested in-silico to explore future possibilities.

A number of earlier published reviews [2–10] addressed some early technical aspects of VMAT or covered the role of VMAT in general or in other districts (lung, head and neck, brain or SBRT). Fiorentino [6] proposed a case review of a bilateral breast irradiation with a limited review of literature, mostly focused on tomotherapy practice.

Aim of this review is to summarize the clinical evidence from literature and provide an overview of the main technical aspects and of the ongoing research activities to consolidate the knowledge about the role of VMAT for breast cancer treatment.

## Materials and methods

### Search and selection criteria

The database of the National Library of Medicine was searched through the Pubmed/MEDLINE service. The time search was limited to articles published after January 2008 when the article of Otto [1] was published. The following keys were searched in all field of the article record: “breast” and any of the following: “volumetric modulated arc therapy”, “VMAT”, “RapidArc”, “Rapid Arc”, “hybrid IMRT” or “hybrid-IMRT” or “hybrid intensity modulated radiotherapy”. The resulting set of references was further pruned after full text examination to exclude irrelevant articles errononeously associated to the selection criteria, duplicate records or reviews.

The time selection was set to the publication of the original article describing the modern concept of VMAT. This intentionally excluded the predecessors like intensity modulated arc therapy (IMAT) and all its variants. Similarly, the literature search exluced from the primary keys the use of Helical Tomotherapy or of its derived TomoDirect (TD) approach specific to breast since the review scope was to discuss the linac-based use of VMAT. Some Tomotherapy related articles remained after the selection being relative to treatment planning comparisons among different techniques. The rational for this choice relies on two arguments. Firstly, IMAT is certainly a predecessor of VMAT but it is a relatively old approach, somehow limited to few institutes because the absence of commercial and broadly available planning system and because its complex clinical workflow. Secondly, for Tomotherapy was excluded because of somehow opposite reasons. Its relatively wide spread and the existing literature is adundant. Including it in this review it would have diluted the focus from a specific technical approach (VMAT) to a more general topic. The deision was not based on the belief that different outcome should be expected when using VMAT or Tomotherapy.

All articles were retrieved from the publisher’s archives and full-text versions used for the data analysis.

### Data analysis

All the publications were tagged according to two main categories: treatment planning studies or clinical reports. Within each category, sub-classes were identified according to some relevant technical or clinical features. Each article was allowed to belong to multiple sub-classes but only to one main category.

The sub-classes were defined as: estimates of secondary cancer risk (RE), accelerated partial breast irradiation or boost use of VMAT (APBI/boost), post mastectomy patients (PM), nodal irradiation (Nodal), bilateral breast irradiation (BiB), respiratory gating (GAT), simultaneous irradiation boost (SIB), single fraction (SF), use of flattening filter free photon beams (FFF), prone positon (PP), hybrid techniques and alternative techniques (Alt/Hybrid), optimisation special recommendations (Optim), helical tomotherapy (HT), knowledge based planning (KBP).

## Results

The general selection resulted in 131 candidate articles, of these, 64 were rejected because of several reasons. These included: different topic within or even outside the VMAT arena or generic studies in VMAT not focused on breast, case reports, reviews, different techniques. Of the remaining 67 publications, 6 belonged to the clinical category and 61 to the treatment planning group.

### Clinical studies

**Fig. 1:**
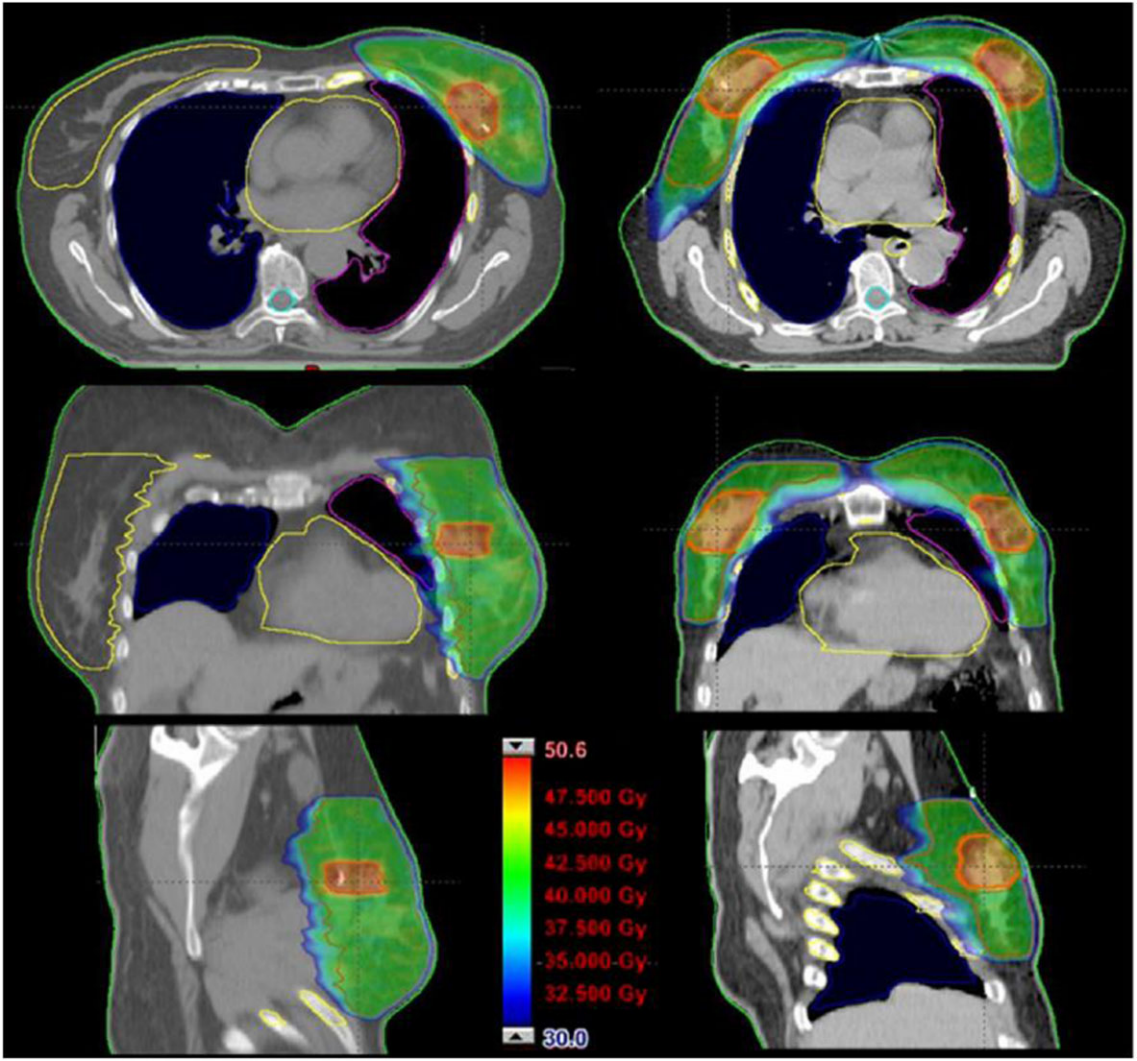
Examples of achievable dose distributions for uni- and bi-lateral breast cancer VMAT treatments with VMAT

The six publications reporting some clinical data [11–16] included only two studies evaluating a prospective phase I-II trial on the use of VMAT with an accelerated SIB fractionation. Table 1 summarizes the clinical studies in a synoptic view. Fig. 1 shows examples of achievable dose distributions for uni- and bi-lateral breast cancer treatments with simultaneous integrated boost and VMAT.

**Table 1:** Synoptic view of the clinical articles

Ref. #	Authors	Number of patients	Type of treatment	Median follow-up	Toxicity
[11]	Scorsetti et al.	50	SIB, 40.5Gy to whole breast and 48Gy to tumor bed	12 months	Skin: Max G3 (1 case) No other toxicity
[12]	De Rose et al.	144	SIB, 40.5Gy to whole breast and 48Gy to tumor bed	37 months	Skin max G3 (1 case) Lung: max G1 (36 cases) No other toxicity
[13]	Riou et al.	9	APBI, 40 Gy in 4Gy fractions twice a day over 5 days.	26 months	Acute: Max G1 Late: Max G1 (inclusive of skin, pneumonitis,pain, oedema)
[14]	Kim et al.	31	VMAT with nodal involvement (internal mammary chain)	25.2 months	Late max G2 No cardiac toxicity
[15]	Lauche et al.	HT: 31 VMAT: 42	SIB. Tumor bed: 63.2-63.8Gy; whole breast: 52.2Gy; supraclavicular nodes: 50.4Gy; internal mammary chain nodes: 52.2Gy	Not explicitly reported. 3 months assumption from toxicity assessment statement	Skin: Max G3 (5% of patients, irrespective of technique) Oesophagus: Max G2 (35-40%) No lung toxicity
[16]	Fiorentino et al.	16 patients	SIB for synchrounous bilateral breast. 50Gy in 25 fractions to the whole breast, 60Gy to the tumor bed.	24 months	No G3 of any type Max G2 acute or late skin toxicity Max G1 acute dysphagia.

*HT* helical tomotherapy, *VMAT* volumetric modulated arc therapy, *APBI* accelerated partial breast irradiation, *SIB* simultaneous integrated boost

In the original study, Scorsetti et al. [11] reported about a 3 weeks accelerated course with 40.5Gy to the whole breast and 48Gy to the tumor bed. Fifty patients were treated with the RapidArc (Varian Medical Systems, Palo Alto, USA) version of VMAT. This was an interim report of a prospective phase I/II trial. With a relatively short median follow-up of 12 months, the maximum acute skin toxicity by the end of treatment was grade 0 in 20/50 patients, Grade 1 in 32/50, Grade 2 in 0 and Grade 3 in 1/50. Three of the treated patients were bilateral cases.

In the most recent report, De Rose et al. [12] updated about 144 patients with at least 2 years of follow-up were treated according to the same protocol. These patients had a median follow up of 37 months. Four of those patients were treated for bilateral breast cancer. No cases of acute skin toxicity of G3 or more were observed during the treatment and the highest reported was G2, with the highest frequency (8%) during the third week of treatment. The only case of G3 was observed at 1-month after treatment (a bilateral patient) and recovered within 3 months. At 1 year the highest reported skin toxicity was G1 (dermatitis) in 14% of the patients, reduced to 4% at the last followup. A correlation between late skin toxicity and the breast volume receiving more than 36.45Gy (*p* < 0.0001) was reported. About lung toxicity, only a radiographic evidence of some change in the lung texture was recorded in 25% of the cases with a maximum of G1 of pulmonary fibrosis. No correlation was found against dosimetric data. No heart toxicity was observed within the follow-up period. Breast pain was reported in 3.5% of the patients at the last follow-up starting from 21.6% at 6 months. Liponecrosis was observed in 23.4% of the patients mainly localized in the boost region. Regarding tumor control, 143 patients had no recurrence while 1 patient developed distant metastases at 39 months after radiotherapy.

Riou et al. [13] reported the use of APBI in an elderly population. Nine patients with a median age of 74 years were treated with 40Gy prescribed in 4Gy fractions twice a day for 5 days. No acute or late toxicities more severe than G2 were observed with no replapses over a median follow-up time of 26 months. The target included the clinical target volume, CTV, plus an isotropic margin of 18 mm to define the planning target volume, PTV. One patient was treated for bilateral disease.

Kim et al. [14] discussed about the use of VMAT in patient requiring internal mammary nodes irradiation over a group of 31 patients with a median follow-up of 25.2 months. The incidence of G2 or more lung toxicity was 3.2% while no clinically overt cardiac toxicity was observed. Skin toxicity was not reported.

Lauche et al. [15] compared clinical data from treatments performed either with linac based VMAT or HT. In this dataset, 31 patients were treated with HT and 42 with VMAT. All the patients were treated with SIB with different dose levels: 63.8 Gy (HT) and 63.2 Gy (VMAT) for the tumor bed; 52.2 Gy in the breast and 50.4 Gy in the nodal region (HT) or 52.2Gy and 49.3Gy for VMAT. The dosimetric findings reported in the study demonstrated a substantial equivalence of the two techniques. Acute skin toxicity of G3 was reported in 5% of the patients regardless of the technique. No lung toxicity was observed. Reported data was based on clinical examinations up to 3 months after treatment.

Fiorentino [16] summarised the findings of their activity on bilateral breast patients. Sixteen patients received VMAT treatment with SIB to the whole bilateral breasts. A dose of 60.0Gy was prescribed to the tumor bed and 50Gy to the whole breast, all in 25 fractions. With a median follow-up of 24 months, no acute or late side effects more than G2 were reported (mostly skin toxicity). No patient relapsed at the last follow-up.

Although very limited and still with short follow-up, all the clinical data reported suggest that the use of VMAT for the treatment of breast cancer is feasible, also in challenging situations such as bilateral targets or patients requiring nodal irradiation. The toxicity profiles reported are modest and compatible with the results reported with other techniques. The need of long follow-up, particularly for endpoints such as cardiac toxicity and second cancer risk is obvious to demonstrate the long term outcome and larger sets of patients would be desirable.

### Treatment planning studies

The 61 studies [17–77] investigating treatment planning issues are listed in alphabetical followed by date of publication order. Table 2 allows navigation of the references according to the sub-classes. The message obtainable by these experiences is quite consistent with few exceptions. VMAT allowed to improve or equate the level of conformal avoidance achieved with other techniques with a general trend towards reduction of the estimated treatment time and monitor units. The division in sub-classes allows to better appraise the specific messages.

- *Dosimetric perspective*:

**Table 2:** Navigation view of the planning investigations (not included the clinical references approaching some of the sub-classes)

Sub-class	References
Risk analysis	[17, 25, 34, 37, 41, 69]
Nodal involvement	[22, 24, 52–55, 59, 63, 67, 75, 77]
Post Mastectomy	[26, 45, 49, 59, 62, 64, 69, 71, 75, 76]
SIB	[19, 21, 23, 25, 29, 38, 47, 48, 50, 63, 67, 70]
APBI/Boost	[18, 27, 28, 42, 56, 57, 60, 62]
Bilateral breast	[29, 50]
Prone position	[42, 73, 74]
Single fraction	[23, 73]
Flattening filter free beams	[21, 25, 40, 47, 61, 62]
Planning strategies	[22, 30, 31, 48, 53, 66, 68, 74]
Hybrid technique	[19, 21, 35, 38, 43, 61]
Alternative approaches	[28, 39, 42, 45, 46, 56, 60]
Respiratory Gating	[27, 35, 51, 52, 55, 64]
Comparison with helical tomotherapy	[32–34, 49, 58]
Other general	[20, 36, 39, 43, 44, 46, 65, 72]

*APBI* accelerated partial breast irradiation, *SIB* simultaneous integrated boost

Breast radiotherapy can include several different targets, depending on the stage, nodal involvement and/or surgical intervention. These include the whole breast, the tumor bed, the chest wall in the post-mastectomy patients, the nodal regions (internal mammary and supraclavicular stations), only to cite some of them. All of these conditions were investigated comparing VMAT against several photon based techniques. In particular, the role of VMAT when nodal irradiation is required was investigated by [22, 24, 52–55, 59, 63, 67, 75, 77]. The post-mastectomy situation was studied by [26, 45, 49, 59, 62, 64, 69, 71, 75, 76].

The general trend reported by the majority of the studies can be summarised in a few key conclusions: i) target coverage, homogeneity and conformality are in general good to optimal; ii) sparing of ipsilateral organs at risk can be equivalent to what achievable with other techniques or further improved; iii) the use of high intensity photon beams, partial arcs and special field arrangements all contribute to the possibility to use of VMAT in all the clinical treatment situations; iv) VMAT, with today’s technical implementations, is generally much simpler and faster to deliver compared to IMRT for the clinical situations encountered with breast cancer.

Where most of the planning studies compared different linac-based techinques, an interesting sub-class of studies presented comparisons of VMAT with respect to either HT or its “fixed” mode, TomoDirect (TD) [32–34, 49, 58]. Also the clinical study from Lauche [15] contributes to the discussion. Qi [58] suggested that VMAT or HT resulted dosimetrically preferable to IMRT or TD but without definitive preference. Nichols [49] studied 15 patients and compared VMAT to HT. Both techniques provided clinically acceptable plans with different features (VMAT improved conformality and organs sparing at lower doses while HT results in better homogeneity and better organs sparing at high doses). Authors concluded stating that both techniques were clinically used in their institute. Haciislamoglu [32, 33] showed with two studies on 15 and 10 patients that both VMAT and HT were dosimetrically comparable with HT slightly improving the medium-high dose sparing for the organs at risk but increasing the low dose spread to the healthy tissues. Han [34] in their risk estimation studies (discussed below) concluded that the TD approach resulted preferable to the others. All in all, including the clinical reports, no study proved an absolute differential benefit from one technique or the other suggesting a fair equivalence between the two approaches from a dosimetric perspective.

To conclude this section, it is worth to mention two negative studies. Badakhsi [20] concluded that the hypothesis of equivalence between VMAT and IMRT was negated by the in-silico data from 12 patients. This was observed for both target coverage and organs at risk sparing, especially for low dose levels (V_2Gy_, V_5Gy_ and V_10Gy_) and for mean doses.

Similarly, Jin [36] demonstrated on 20 left-sided patients that VMAT was inferior to IMRT in terms of target coverage and low-dose organs at risk sparing and did not recommended VMAT for these patients.

- *Secondary cancer and risk estimates*:

Six publications were tagged within this sub-class [17, 25, 34, 37, 41, 69].

Abo-Maydan [17] suggested that VMAT might have a higher risk of secondary cancer induction compared to conformal or tangential IMRT. Data are based on 10 patients.

Dobler [25] summarized for 6 patients the excess absolute risk for second cancer for right-sided breast patients to be treated with SIB and either flattened (FF) or Unflattened (FFF) photon beams. Their data showed a significant reduction of the risk to the contralateral and peripheral organs when FFF beams were used in conjunction with a tangential arcs approach. The risk reduction amounted to ~25-29% or 44-58% if tangential FFF VMAT was compared to tangential FF or full arc or IMRT approaches.

Han [34] compared for 10 patients five different approaches (from conformal therapy to HT in the TD form) and the lifetime attributable risk (LAR) resulted the worst for VMAT while TD scored best for most of the organs considered in the analysis. The main concern about this study is that the linac-based plans were all computed using a very old version of the VMAT software, basically the first generation of it, and no evidence of any strategy for the minimisation of the uninvolved contralateral organs was provided.

Johansen [37] computed in 8 patients the estimated excess relative risk (ERR) with linear and non linear models for conformal RT, IMRT and VMAT data. The VMAT plans resulted with an intermediate non-linear ERR (0.33 compared to 0.31 for conformal and 0.39 for IMRT), with no statistically significant difference.

Lee [41] compared conformal, IMRT and VMAT approaches measuring phantom doses with photoluminescent detectors and demonstrated that the LAR of secondary malignancies was lowest for conformal RT and maximal for IMRT with VMAT falling inbetween the two.

Wang [69] computed for 30 patients the tumor control and normal tissue complication probabilities for conformal, fixed field tangential IMRT and single arc VMAT. Their results suggested that TCP and NTCP for target volume and left lung, respectively, did not show significant differences. Heart NTCP ranged from 3 to 7% among techniques (5% for VMAT).

With the exception of [37] and to some extent [69], the studies demonstrated a general trend towards somehow higher risk of secondary cancer induction or lifetime risk if VMAT is applied compared to conformal therapy or tangential IMRT. Unfortunately no study of secondary risk is available for VMAT data planned with highly intensive OAR sparing methods and compactified dose distributions as would be available today. Long term clinical data and better designed in silico studies on large cohorts of patients would be needed.

- *Altered fractionations*:

The use of VMAT for APBI or for the boost treatment was reported in 7 articles [18, 27, 28, 42, 57, 60, 62, 65] plus the previously mentioned clinical study from Riou et al. [13]. Since its early days, VMAT was compared against electron, photons and even protons for the treatment of the boost volume. Toscas [65], on 14 patients, seeded the field and demonstrated that VMAT (or IMRT) provided the best coverage of the targed, compatible with protons while enabling the maximal sparing of the dose to the skin (5.4Gy in average compared to 7.2Gy for electrons or 5.8 to 7.5 for all other techniques with photons or protons). The concept was naturally extended to APBI [13, 27, 28, 42, 56, 60]. Popescu [56] interestingly proposed to combine the VMAT approach to the simultaneous rotation of the couch to further boost the sparing of the healthy tissues. Riou’s study [13] is the only one reporting about clinical treatments.

Charaghvandi [23] and Yoo [73] investigated the use of VMAT for single pre-operative fraction delivery. In the first study [23], the tumour of 20 patients with early stage cancer was contoured on a pre-operative MR and contrast-enhanced CT scans. A single dose of 15Gy was prescribed to the clinical target volume and 20Gy to the gross tumor volume. The VMAT plans were compared against interstitial multicatheter brachytherapy. The authors conclude that both approaches could be dosimetrically appropriate but with a preference to VMAT due to the target overdosage inherent to the brachytherapy technology. In the second investigation [73], the authors applied different techniques (conformal, coplanar and non-coplanar IMRT and VMAT) to 16 patients. The dose prescription was 15Gy to the clinical and 18Gy to the gross target volumes. Though VMAT resulted the potentially fastest technique (7 min of beam on time compared to 8.3-11.0 for the others), the authors found a benefit of IMRT in terms of target homogeneity and conformality as well as for skin sparing.

The SIB approach was discussed at various levels in [19, 21, 23, 25, 29, 38, 47, 48, 50, 63, 67, 70].

- *Altered patient positioning and bilateral breast*:

Three studies investigated the case of prone positioning [42, 73, 74] while two planning studies addressed the bilateral breast problem [29, 50] in addition to the clinical studies [11–13, 16].

The prone positioning has been appraised by some authors in conjunction with other innovative aspects. Yoo [73] proposed it for the case of single fraction delivery in partial breast treatments. Sixteen patients were simulated and, compared to other techniques (conformal or fixed field IMRT), VMAT offered the shortest estimated treatment delivery time and better sparing of normal tissue except skin, but yielded less dose conformity and homogeneity within target. For this latter reason, authors concluded that their preference went to fixed field IMRT. Yu [74] studied 10 patients positioned prone to demonstrate the feasibility of small-arc VMAT and concluded that it was possible to improve conformity, homogeneity and dose to organs at risk. Liang [42] investigated for 10 patients in prone position the possibility to use nonisocentric trajectories to deliver modulated arcs for APBI. They concluded that a marked reduction of the irradiation of the uninvolved breast tissue was achievable with this approach.

The number of synchronous bilateral breast patients in routine clinical practice is not too large. For this reason, offering an optimal treatment to these patients might require advanced planning skills. Nicolini [50] published in 2009 the seminal work in this area simulating 10 patients. A SIB fractionation (with different dose levels between left and right breasts if needed) was applied. With a technology still in its infancy, authors showed that VMAT reduced the V_20Gy_ below 10% and MLD < 10Gy and the mean dose to heart to 6Gy (compared to 7.4 for IMRT). This study proved the feasibility of VMAT for bilateral breast treatments, with a single isocenter and the use of asymmetric arcs. The clinical practice reported above reflected this initial pivotal project. More recently, Fogliata [29], in the framework of a knowledge based planning automation project, showed that models developed to optimise unilateral breast treatment, could be applied successefully, with multicentric validation, also to bilateral breast targets. This further simplifies the technical burden connected to the relatively rare incidence of these cases.

- *Alternative techniques or hybrid techniques*:

Hybrid techniques, mixing IMRT with fixed fields and VMAT were reported in 6 studies [19, 21, 35, 38, 43, 61]. The basic rationale of hybrid techniques is to mix fixed beam IMRT to VMAT with the aim of obtaining a benefit from both techniques and mitigate their eventual pitfalls. Aly [19] proposed the use of IMRT fields for the whole breast and VMAT for the boost and studied the method on 12 patients with SIB fractionation. The hybrid VMAT approach outperformed the full VMAT method for both ipsilateral lung and heart (in left-sided patients) dose sparing. Bahrainy [21] reported about an in-silico investigation on 10 patients (left-sided) where two tangential IMRT fields were combined to one VMAT arc. The study was performed for both FF and FFF photon beams. Authors reported that the combination of their hybrid approach to the FFF technology enabled a substantial reduction of treatment time and improved dosimetric potential, all suggesting the applicability of the method for hypofractionated dose schemes.

Other more forward-looking alternative technical approaches were investigated in 7 articles [28, 39, 42, 45, 46, 56, 60]. In most of these studies, not-clinically available strategies were tested. These included non-isocentric trajectories, simultaneous couch and gantry rotations or burst technique (a kind of “step-and-shoot” VMAT where dose is delivered with static gantry at fixed intervals during arc rotation). Ma [46], in opposition to the mainstream of increased dose rate with FFF beams, analyzed with Monte Carlo simulations, the possibility to deliver kind-of pulsed VMAT with low dose rates (0.2Gy/arc with 3 min of interval between arcs to achieve an effective dose rate of 0.67Gy/min) All these methods require dedicated and specialised planning and delivery technology not commonly and not clinically available today.

- *Respiratory Gating*:

In six publications [27, 35, 51, 52, 55, 64], the use of respiratory gating was specifically addressed. The first article was published by Nicolini [51] with a pre-clinical investigation showing a high reliability of the delivery systems to the beam hold required by the gating process, even when high frequency interruptions (free breathing rather than breath hold) were simulated. Among the most recent data, Pham [55] demonstrated that, compared to gated IMRT, gated VMAT might contribute to further reduce mean heart dose in selected patients. On the contrary, Jeulink [35] showed that, under free breathing conditions, hybrid IMRT approaches might be preferable to VMAT in breast treatments.

- *Flattening filter free photons*:

The use of high intensity photon beams was addressed in 6 studies [21, 25, 40, 47, 61, 62] plus the two clinical reports from the Humanitas Cancer Center group [11, 12]. The treatment of the whole breast or of the chest wall, as well as the use of conventional or of SIB fractionation schemes were appraised. In all cases, the data suggested a potential benefit in the use of FFF beams particularly for the SIB schemes. Even in the case of chest wall irradiation [40, 62], the use of FFF could contribute to minimize the dose to the contralateral organs as a consequence of reduced scatter.

- *Special optimisation suggestions or guidelines*

Mancosu et al. [48] discussed the use of automatic constraints on the monitor units showing a possible correlation between the increase of MU and the increase of OAR involvement while modest impact was observed on target coverage. Nicolini et al. [31] investigated and proposed a practical solution to account for the “missing fluence” outside the body outline in the absence of automated “skin flash” tools in the optimisation of VMAT plans. The methodology proposed should compensate for potential underdosage of the most superficial region due to tissue expansion or displacement of the body (breathing, movments, oedema or other mechanisms).

Originally, VMAT was developed and tested aiming to use full arcs, one or multiple. In the case of breast treatments, this might result dosimetrically sub-optimal (mainly due to the involvement of the contralateral structures but also of the ipsilateral lung and heart for the left-sided patients)) if fluence cannot completely go down to zero during full arc treatments because of technical constraints in the optimization process. The use of split or short arcs or the use of avoidance sectors, mimicking the tangential beam concept, was investigated by several groups [22, 30, 53, 66, 68, 74]. All significantly reduced the dose to all the organs at risk while preserving adequate target coverage. Fogliata [30] showed that, either using partial arcs or using “avoidance” sectors (basically dropping the dose rate to 0 within sub-arcs sectors, dosimetrically equivalent to the use of split arcs but technically easier), the mean dose to the heart was reduced by 51%, 12% for the ipsilateral lung, by 81% for the contralateral lung and by 73% for the contralateral breast compared to full arcs. All differences were significant with *p* < 0.001. The absolute mean dose to the heart (left treatments) was dropped to <2Gy; the mean dose to the contralateral lung as well as to the contralateral breast to ~0.6Gy for hypofractionated treatments of 40.05Gy in 15 fractions.

## Discussion

Despite VMAT having been introduced in clinical practice approximately 10 years ago and despite its wide application in many different clinical indications [2–10], its application to the treatment of breast cancer is still limited. This is reflected in the paucity of published reports on clinical experiences.

### Clinical studies

Concering the clinical reports [11–16], few general factors can be outlined. The short follow-up of these studies limits the evidence mostly to the area of acute toxicity which was reported to be very modest by all authors. Late toxicity and control (up to 2 years) data are promising but of course cannot be conclusive. The main limit consisted in the small sample of patients reported. The exception comes from the only one prospective phase I/II trial has been reported so far [11, 12], with the latest interim analysis reporting, data of 144 treated patients with a minimum follow-up of 2 years. This is the largest sample so far. This protocol combined also two important clinical themes: the use of SIB fractionation and accelerated treatments, uni- or bilateral treatments. The data from all studies are basically consistent and encouraging. For example, Kim et al. [14] and Lauche et al. [15] demonstrated, although with smaller samples, the possibility to achieve good results for the lymphnodal irradiation with VMAT, a result quite challenging with other techniques. Of course larger cohorts, longer follow-up and more structured trials are needed in this respect.

It is nevertheless reasonable to believe that the actual clinical adoption of VMAT for breast is wider.

### Ttreatment planning studies

the wide adoption of VMAT for breast, might be extrapolated from the extensive literature published about in-silico investigations covering basically all technical and clinical areas [11–77]. From the comprehensive appraisal of this category and its sub-classes, a number of global messages can be derived.

Firstly, and unfortunately, also the merely planning studies are frequently limited by the small number of patients included. With the exception of the knowledge based planning study [29] where in total more than 200 patients were considered between training and validation phases, the typical sample size in the planning investigations ranges from few units to some tens, unbalanced towards the lower limit. Although the scope of in-silico studies is the proof of the principle, these investigations might benefit from a stronger accounting for the inter-patient variability with more statistical power.

Dosimetric perspective, altered fractionation:, flattening filter free photons: Beside this limitation, the evidence derived from these studies suggests that, from a dosimetric, a deliverability and a logistics point of view, VMAT might be considered for the treatment of the whole breast or for the post mastectomy cases as well as for the patients with or without nodal involvement; also partial breast irradiation was considered and proved to be feasible. Altered fractionation schemes (like SIB, hypofractionation or even single fraction) could be effectively proposed in addition to conventional fractionation. This is a really appealing possibility in general, consistent with many recent recommendations and applicable, in the extreme modalities, to well selected groups of patients. The use of advanced photon beams (the high intensity or flattening filter free beams) was tested and the results suggest that improved sparing of organs at risk, particularly at low doses can be achieved while facilitating delivery of multi-level doses to the targets.

### Special optimisation suggestions or guidelines

Technical recommendations on special optimisation strategies were reported. From these, the use of multiple partial arcs or the use of hybrid VMAT or the combination of both can be considered today as a possible strategy for breast. One of the main topics of breast irradiation is the management of respiratory induced motion. Gated delivery was investigated with success and, as for IMRT and conventional treatments [78], the use of deep inspiration breath hold seems to be the most appropriate approach. One unsolved issue is the relevance of prone compared to supine positioning. Not enough literature was published in this area to draw any conclusion or recommendation. We have not addressed in detail a comparison of the various technical aspects of the arc arrangements (number and length of the arcs, collimator angles, couch rotations) since this would be challenging in a synoptic view doe to the excessive number of variants. Readers are referred to the original publications for these details.

### Secondary cancer and risk estimates

While in conclusion from a dosimetric/logistics point of view VMAT seems to be comparable with other modulated strategies, two delicate issues remain for all isotropic treatment strategies, trading reduced volumes treated to high doses with larger volumes treated to lower doses. One is the risk of secondary malignancy induction in a group of patients with a general long life expectancy. While modelling currently suggests a potential slight increase in second cancer risk with IMRT/VMAT, recent clinical data in prostate cancer [79] and preclinical data [80, 81] suggest that the opposite might be the case because the second-cancer-risk/dose relationship may actually be supralinear with animal data actually suggesting a threshold-like-dose of >25 Gy for single dose exposure.

Moreover, a topical systematic review on dose-response relationships for solid cancers induction in humans showed that the excess risk per Gy is considerably lower after fractionated radiation therapy than after acute lower-dose exposure experienced by the Japanese atomic bomb survivors, in the range of 5-fold to 10-fold lower, and that at higher fractionated doses it is unlikely that the risk would decrease (with the exception of thyroid cancer) [82]. Old models used for secondary cancer risk estimates may have not included these important changes, and would generally end up in a theoretically negative impact of VMAT in comparison with 3D-CRT. Recent modelling studies on breast cancer risk associated to mediastinal VMAT in Hodgkin’s lymphoma did not show any additional risk in comparison with 3D-CRT when appropriate dose constraints are used [83]. The hypothesis of reduced second cancer risk with modulated techniques has been outlined conceptually recently [84].

### Cardiac toxicity

The second issue is cardiac toxicity. Meaningful epidemiological data regarding cardiac toxity from radiotherapy exist only for tangential radiotherapy. As it has recently been pointed out, these data cannot be used to estimate risk from dose distributions with a different character using an appropriate biological model [85] a quasi-threshold might exist also in this area and recent imaging data from clinical series [86] have in fact suggested that such a threshold (20-30 Gy) as predicted by the models may exist, again resulting in a favourable outcome with modulated techniques such as VMAT.

In summary, regarding those two issues,in the absence of strong positive or negative evidence, some caution in the selection of patients is advisable. Simple cases with no heart exposure and minimal lung exposure upon tangent treatments, might not benefit from VMAT while the more complex situations, due to anatomy or dose prescription or other concomitant reasons, could likely benefit in terms of clinical estimates from VMAT. The latter group, could include large breasts, highly concave chest walls as anatomical features and particularly the treatments that include draining lymph nodes.

## Conclusions

The role of VMAT in the radiation treatment of breast cancer seems to be consolidated in the in-silico arena while still limited evidence and only one phase II trial appeared in literature from the clinical viewpoint. More clinical reports are needed to fully proove the expected dosimetric benefits demonstrated in the planning investigations.

## Abbreviations

Alt/Hybrid: Alternative/hybrid
APBI: Accelerated partial breast irradiation
BiB: Bilateral breast
CT: Computed tomography
CTV: Clinical target volume
FF: Flattened
FFF: Flattening filter free
GAT: Gating
HT: Helical tomotherapy
IMAT: Intensity modulated arc therapy
IMRT: Intensity modulated radiotherapy
KBP: Knowledge based planning
LAR: Lifetime absolute risk
LC: Local control
MU: Monitor units
NTCP: Normal tissue complication probability
OAR: Organ at risk
Optim: Optimisation
OS: Overall survival
PM: Post mastectomy
PP: Prone position
PTV: Planning target volume
RE: Risk estimate
RT: Radiotherapy
SBRT: Stereotactic body radiation therapy
SIB: Simultaneous integrated boost
TCP: Tumor control probability
TD: Tomo direct
VMAT: Volumetric modulated arc therapy
